# Changes in pulmonary tuberculosis prevalence: evidence from the 2010 population survey in a populous province of China

**DOI:** 10.1186/1471-2334-14-21

**Published:** 2014-01-11

**Authors:** Xiaolin Wei, Xiulei Zhang, Jia Yin, John Walley, Rachel Beanland, Guanyang Zou, Hongmei Zhang, Fang Li, Zhimin Liu, Benny CY Zee, Sian M Griffiths

**Affiliations:** 1 Chinese University of Hong Kong, Hong Kong, China; 2 Shenzhen Municipal Key Laboratory for Health Risk Analysis, Shenzhen Research Institute of The Chinese University of Hong Kong, Shenzhen, Guangdong Province, China; 3 Center for Tuberculosis Control, Shandong Provincial Chest Hospital, 12 Lieshishan Dong Lu, Jinan 250101, China; 4 Nuffield Centre for International Health and Development, University of Leeds, Leeds, UK

**Keywords:** Tuberculosis, Population based prevalence survey, Case finding, China

## Abstract

**Background:**

This paper reports findings from the prevalence survey conducted in Shandong China in 2010, a province with a population of 94 million. This study aimed to estimate TB prevalence of the province in 2010 in comparison with the 2000 survey; and to compare yields of TB cases from different case finding approaches.

**Methods:**

A population based, cross-sectional survey was conducted using multi-stage random cluster sampling. 54,279 adults participated in the survey with a response rate of 96%. Doctors interviewed and classified participants as suspected TB cases if they presented with persistent cough, abnormal chest X-ray (CXRAY), or both. Three sputum specimens of all suspected cases were collected and sent for smear microscopy and culture.

**Results:**

Adjusted prevalence rate of bacteriologically confirmed cases was 34 per 100,000 for adults in Shandong in 2010. Compared to the 2000 survey, TB prevalence has declined by 80%. 53% of bacteriologically confirmed cases did not present persistent cough. The yield of bacteriologically confirmed cases was 47% by symptom screening and 95% by CXRAY. Over 50% of TB cases were among over 65’s.

**Conclusions:**

The prevalence rate of bacteriologically confirmed cases was significantly reduced compared with 2000. The survey raised challenges to identify TB cases without clear symptoms.

## Background

China, with an estimated prevalence of all TB cases of 108 per 100,000 in 2010, has the second highest TB burden in the world, accounting for 13% of all cases worldwide [1]. The World Health organization (WHO) estimated that China had reached the targets of 85% treatment success by 1993 and 70% case detection rate by 2005 [2]. National TB prevalence surveys were conducted in China in 1979, 1990, 2000 [3], and 2010 [4]. Survey results provide more accurate estimates for TB prevalence rates than the WHO estimates and can be used to assess the likelihood of China achieving global targets for TB prevalence.

Shandong province has a population of 94 million. It is a relatively developed province with a per capita GDP 1.6 times of the national average in 2010 [5]. The prevalence rate of TB in Shandong was lower compared with the average rate of China in 2000 [3]. Population representative samples were drawn in Shandong in the surveys of 2000 and 2010 using similar methods. The study aimed to estimate the TB prevalence in Shandong based on the 2010 survey, and compare results of the two cross sectional surveys.

## Methods

### Study population

The target population of the TB prevalence survey was residents of 15 years old or above who had lived in the selected clusters for more than 6 months. A population based, cross-sectional survey was conducted using multi-stage random cluster sampling method.

The survey employed the same sampling methods as the China national survey in 2010, which was similar to the sampling methods used in 2000 [6]. The design of the surveys was in accordance with WHO recommendations [7]. The design effect factor due to cluster sampling was estimated at 1.28 [8]. A sample size of 52500 adults (≧15 years old), in 35 clusters, was calculated based on detecting a change of 20% in prevalence rate of TB smear positive cases compared with the rate of the 2000 survey (95 per 100,000), with a probability greater than 95% and 95% power, accounting for 90% response rate of participants [9].

A stratified multi stage random sampling was used to select the 35 clusters within 17 prefectures in Shandong province. The number of clusters was randomly allocated in proportion to the provincial population at the prefectural, county/district and township levels. A cluster was defined as a community (a village in the rural area or a resident community in an urban area) with a population of 1250 to 1750 adults (i.e., those of 15 years or older). If the community contained less than 1250 adult residents, the neighboring community to the north was annexed. If the community or combined communities containing more than 1750 adults, we randomly selected households and then included all adults in the household for the survey until the total number of selected adults reached 1750. Military barracks and prisons located in the cluster were excluded [7].

### Data collection

The survey was conducted from March to June 2010 by survey teams consisting of clinicians, public health doctors, radiologists, laboratory technicians and nurses. Local media was used to promote awareness of the survey. Community workers conducted a house-to-house census to update the database of residents, inform survey participants and obtain informed consent. The study did not involve children under 15 years old. Written informed consent was obtained from all participants of 16 years old or above. While from those of 15 years old, written informed consents were obtained from their parents or guardians. All documents were properly stored in the Shandong Chest Hospital. Ethical approvals for the study and consent procedures were obtained from the Institutional Review Board (IRB) of Shandong Chest Hospital (NIH register numberIRB00006010).

Those who agreed to participate in the survey were invited to the county TB dispensary, where they completed a consultation with a trained clinical TB doctor regarding any symptoms suggestive of TB, such as persistent cough (lasting two weeks or longer), haemoptysis, weight loss and fever. All participants had a chest X-ray (CXRAY) taken that then were reviewed by a panel of radiologists. Those with symptoms or CXRAY films suggestive of TB were classified as suspected TB cases. All suspected cases were asked to produce three sputum samples, one at the time of consultation, another at night and the third in the early morning of the following day. Identified suspects completed an additional questionnaire regarding their social-economic situation, smoking status, and the presence of TB related symptoms in the preceding six months (cough, fever, weight loss, chest pain and haemoptysis).

### Classification and quality control

Sputum smears were conducted in local TB dispensaries. All sputum samples were cultured using the Löwenstein–Jensen medium in the provincial laboratory within 24 hours using cold chain transportation. Samples were excluded from TB when non-tuberculosis bacilli were identified from the culture. All sputum smear and culture were conducted strictly according the national TB laboratory external quality control measure, which is in consistent with the WHO TB prevalence survey guideline [7]. TB classification was made according to the China national TB guideline [10]. A positive smear had at least one acid fast bacillus identified during examination of at least 100 fields. Participants with positive sputum smear specimens were classified as sputum positive cases. Those with positive smear or culture sputum specimens were classified as sputum bacteriologically confirmed cases. Those being culture negative with abnormal CXRAY suggestive of TB and having been ruled out from other diseases by clinicians and radiologists were classified as CXRAY suggestive bacteriologically negative cases. Due to resource limitations the recommendation of broad-spectrum antimicrobial agents to confirm the diagnosis of negative TB cases was not applied in this survey [11]. Newly diagnosed cases were distinguished from previously diagnosed cases through checks during the interviews and against the TB registration system.

Initial diagnosis was made by a group of local clinicians and radiologists. Subsequently, samples and CXRAY films of all suspected and confirmed cases were re-assessed by a group of senior clinicians and radiologists at provincial and national levels. CXRAY films of 100% of those scored as abnormal and 10% random sampling of those scored as normal were transferred for independent reading. The provincial laboratory team randomly examined one slide from the three samples of sputum positive cases, all three samples of CXRAY suggestive TB cases, and randomly selected 10% of the non-TB cases.

### Data analysis

Prevalence estimates of sputum positive, bacteriologically confirmed and all TB cases were calculated. In all analyses, population weightings were employed to adjust for the stratified multi-stage sampling design effect [8]. The survey results in 2010 and 2000 were standardized against the age structures of China’s census population in 2010. The 2000 TB prevalence survey included all age groups [12]. The WHO recommended method was used to enable comparison between the two surveys that prevalence rates of child TB were assumed to reduce to the same extent as adult TB from 2000 to 2010 [13]. Subgroup analysis in gender, age groups and urban/rural residence were conducted. Case identification rate was calculated as the number of cases identified by a screening method over all suspected cases found by the method. Yields of the symptom consultation and CXRAY were calculated as a proportion of the total number of bacteriologically confirmed cases.

## Results

**Figure 1:**
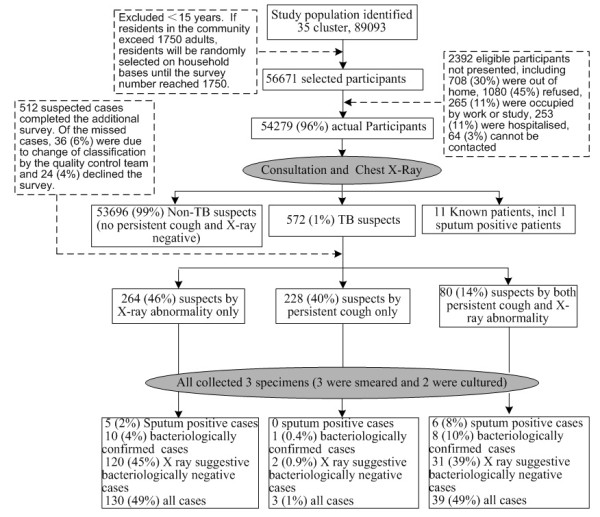
Schematic diagram of data collection in the 2010 tuberculosis prevalence survey in Shandong, China.

The survey selected 17 urban clusters and 18 rural clusters. It covered a total population of 89,093, of which 56,671 were eligible for the survey (Figure 1). The response rate ranged from 95% to 97% in different clusters. 54,279 participants attended clinical consultation and were examined by CXRAY. Among them, 47% were males. The average age was 46 years with 14% of 65 years and older. A total of 572 suspected TB cases were found. Of these, 264 (46%) were identified based on CXRAY abnormalities, 228 (40%) were based on persistent cough, 80 (14%) were based on both. The survey diagnosed 172 new cases, including 19 new bacteriologically confirmed cases (including 11 sputum and culture positive cases, and 8 sputum negative but culture positive cases) and 153 CXRAY suggestive bacteriologically negative cases. The survey also identified 11 existing cases registered on the national TB program. In addition, the survey found four cases with culture positive non-tuberculosis bacilli, and excluded them from TB patients.

### Screening methods

All participants of the survey were first screened by symptoms and CXRAY. Those who had symptoms of consistent cough or haemoptysis, or CXRAY abnormalities were then screened by smear and culture. Case identification rates of new bacteriologically confirmed cases from the suspected cases were significantly higher with CXRAY as a primary tool (Figure 1, 3.8%, P = 0.012) and further increased by both symptom screen of persistent cough and CXRAY (10%, P < 0.001) compared with symptom screen alone (0.4%). The same pattern of case identification rate was observed in the sputum positive cases (7.5%, 1.9% and 0% respectively). The proportion reporting persistent cough was not significantly higher among bacteriologically confirmed cases compared with other suspects (P = 0.565). The symptom consultation alone identified 308 suspects, including 6 (1.9%) sputum smear positive TB and 9 (2.9%) bacteriologically confirmed TB. Among the 344 suspects with CXRAY abnormalities, 11 (3.2%) had sputum positive TB and 18 (5.2%) had bacteriologically confirmed TB. The yield of bacteriologically confirmed cases was 47.4% by screening consultation and 94.7% by CXRAY. In the population of over 65 years old, symptom consultation and the CXRAY identified 174 and 182 suspected cases respectively, yielding5 (2.9%) and 9 (4.9%) of bacteriologically confirmed cases. Yields of bacteriologically confirmed cases were 55.6% by symptom consultation and 100% by CXRAY among over 65’s.

Of the 512 suspected cases that completed the additional questionnaire, 42% were farmers and 31% were current smokers (Table 1). Per capita household income of bacteriologically confirmed cases was less than 50% of that of the non-TB cases (P < 0.05). Though smoking rate was higher among TB cases compared with non-TB cases, no significant differences were found (P > 0.05). Of the ten bacteriologically confirmed cases not presenting with persistent cough at the prevalence survey, one coughed for two days, one had chest pain, and the other eight had no symptoms of TB in the last six months.

**Table 1:** Questionnaire survey results of the 512 TB suspects identified from the TB prevalence survey in Shandong, China

	**Sputum positive cases (%)**	**Bacteriologically confirmed cases (%)**	**All cases (%)**	**Non-TB cases (%)**	**Total**
Total	11 (2)	19 (4)	172 (34)	340 (66)	512
Age					
15-64 years old	5 (45)	10 (53)	73 (42)	147 (43)	220 (43)
65 years and older	6 (55)	9 (47)	99 (58)	193 (57)	292 (57)
Occupation					
Government	1 (9)	1 (5)	9 (5)	30 (9)	39 (8)
Sales persons	0	1 (5)	7 (4)	12 (4)	19 (4)
Migrant workers	0	2 (11)	14 (8)	26 (8)	40 (8)
Farmers	7 (64)	10 (53)	80 (47)	136 (40)	216 (42)
Jobless	3 (27)	5 (26)	49 (28)	91 (27)	140 (27)
Others	0	0	13 (8)	45 (13)	58 (11)
Education					
Illiterate	4 (36)	8 (42)	82 (48)^a^	126 (37)	208 (41)
Under high school	6 (55)	8 (42)	80 (47)	159 (47)	239 (47)
High school and above	1 (9)	3 (16)	10 (6)^b^	55 (16)	65 (13)
Smoking status					
Smokers	5 (45)	7 (37)	56 (33)	102 (30)	158 (31)
Non smokers	6 (55)	12 (63)	116 (67)	238 (70)	354 (69)
Per capita household income (RMB per year)	3297	3374^c^	4971^c^	7272	6495

^a^Illiterates had the highest proportion of TB cases compared with people with other educational backgrounds (x^2^ = 5.336, P = 0.021).

^b^People with high education or above had the lowest proportion of TB cases compared with people with other educational backgrounds (x^2^ = 11.066, P<0.001).

^c^Average per capita income of patients who were bacteriologically confirmedcases (Z = -2.126, P = 0.034) and all TB cases (Z = -3.673, P<0.001) were significantly lower than that of people who did not have TB. (1USD = 6.6RMB in 2010).

### Estimated prevalence

**Figure 2:**
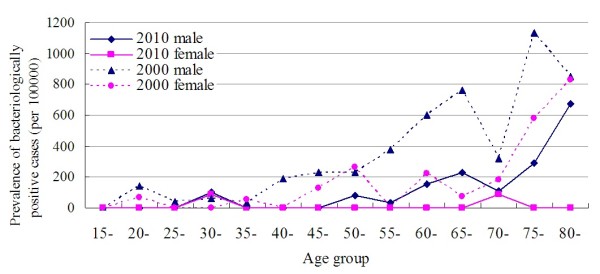
Comparison of prevalence of bacteriologically confirmed cases between 2010 and 2000 by age and gender.

The crude prevalence rate in Shandong in 2010 of sputum positive cases was 22.1 (95% CI: 9.6-34.6), bacteriologically confirmed cases was 36.8 (95% CI: 17.8-55.8), and all cases were 337.1 (95% CI: 254.1-420.0) per 100,000 in adult population (Table 2). The adjusted prevalence rates of the whole population in Shandong were17.8 (95% CI: 8.3-17.5), 27.8 (95% CI: 14.8-28.0) and 239.4 (95% CI: 179.9-298.9) per 100,000 in 2010. A remarkable decline of 82.0%, 80.2% and 31.4% was observed in TB prevalence rates of sputum positive, bacteriologically confirmed, and all cases, respectively, compared to the adjusted rates in 2000 [12]. Large declines were observed in males between 40 and 65 years old, and in females over 60 years old (Figure 2).

**Table 2:** Prevalence rates of sputum positive TB cases, bacteriologically confirmed TB cases and all cases in Shandong, China, 2010

	**No. of participants**	**Rates of sputum positive cases (per 100000)**	**Rates of bacteriologically confirmed cases (per 100000)**	**Rates of all cases (per 100000)**
Crude	54279	22.1 (9.6–34.6)	36.8 (17.8–55.8)	337.1 (254.1–420.0)
Adjusted for all population^a^		17.8 (8.3–17.5)	27.8 (14.8–28.0)	239.4 (179.9–298.9)
Adjusted for adults^a^		21.4 (10.0–32.8)	33.5 (17.8–49.2)	285.8 (215.2–356.4)
Sex: Male^a^	25406	29.5 (7.9–51.1)^b^	43.3 (15.9–70.7)^b^	388.0 (282.2–493.8)^b^
Female^a^	28873	5.4 (0.9–9.9)	15.7 (3.9–27.5)	183.4 (128.1–238.7)
Age: 15-64^a^	46807	9.9 (1.4–18.3)	20.8 (5.0–36.6)	154.8 (113.9–195.6)
65 and older^a^	7472	83.3 (17.2–149.4)^c^	118.3 (35.0–201.7)^c^	1429.9 (1011.4–1848.3)^c^
Area: Urban^a^	26101	9.0 (1.0–17.0)	9.0 (1.0–17.0)	139.1 (80.7–197.5)
Rural^a^	28178	24.2 (4.8–43.6)	43.1 (17.0–69.2)^d^	342.6 (242.4–442.8)^d^

^a^Adjusted for sampling errors and against China’s census population in 2010.

^b^Significantly higher than female of prevalence rates of sputum positive cases (x^2^ = 9.702, P = 0.002), bacteriologically confirmed cases (x ^2^ = 11.722, P = 0.001), and all TB cases (x ^2^ = 32.378, P<0.001).

^c^Significantly higher than year 15–64 of prevalence rates of sputum positive cases (x^2^ = 20.083, P<0.001), bacteriologically confirmed cases (x^2^ = 22.128, P<0.001), and all TB cases (x ^2^ = 307.665, P<0.001).

^d^Significantly higher than urban area of prevalence rates of bacteriologically confirmed cases (x ^2^ = 6.323, P = 0.012) and all TB cases (x ^2^ = 26.904, P<0.001).

The adjusted prevalence rates in the adult population were 21.4 (95% CI: 10.0-32.8), 33.5 (95% CI: 17.8-49.2) and 285.8 (95% CI: 254.2-356.4) for sputum positive cases, bacteriologically confirmed cases and all cases, respectively. Significant differences regarding adjusted TB prevalence rates were observed between males and females, over 65’s and 15 to 64 years old, in rural and urban areas (Table 2, P < 0.001). The male to female ratios were 5.5 in sputum positive cases and 2.8 in bacteriologically confirmed cases, while the ratios climbed to 6.0 and 4.1, respectively, among those over 65 years. The majority of TB patients, 54.5% of sputum positive cases and 47.3% of bacteriologically confirmed cases, were from people 65 years or older. The ratio between over 65’s and 15 to 64 years old was 8.4 in sputum positive cases and 5.9 in bacteriologically confirmed cases. The ratio between rural and urban areas was 2.7 in sputum positive cases and 4.8 in bacteriologically confirmed cases.

## Discussion

The most striking finding was that a large proportion of TB patients did not present consistent cough. Passive case finding is the routine practice in developing countries where sputum microscopy is performed to identify TB cases among people with persistent cough. A large proportion of TB cases may be missed using this method as 53% of bacteriologically confirmed cases and 45% sputum positive cases in this study had no persistent cough but were identified through abnormal CXRAY. Nearly half of bacteriologically confirmed cases reported no symptoms in the last six months. This finding, although initially surprising, is consistent with reports from Vietnam (47% of bacteriologically confirmed cases not presenting persistent cough) [14], Myanmar (38%) and Ethiopia (48%) [13]. CXRAY was sensitive in detecting TB cases, as yields of bacteriologically confirmed cases were much higher by CXRAY compared with by symptom screening, as reported in Vietnam [15] and some high HIV prevalence settings [16,17]. CXRAY, though expensive at the initial installment, may improve TB case finding due to its short turnover time and high throughput [18]. Our findings suggest that the strategy of case finding using CXRAY followed by sputum or culture as the primary and secondary screening tests could be more effective, especially among the population of over 65 year olds, as the yields were higher in over 65’s compared with the general population. Although using CXRAY to examine everyone is not feasible, it can be used in routine elder physical examinations. The China public health package now covers free CXRAY for elders, as well annual employee body examinations provided free CXRAY.

In this survey, only one sputum positive patient had been detected and treated by the national program, though specific clinical consultation was conducted to identify any patients who have been diagnosed and treated for TB before. This may reflect the difference between the active case finding approach in the survey and the passive casing finding approach in practice. Nevertheless, it indicated that a large proportion of bacteriologically confirmed TB cases are missed by the national TB program.

Another notable change is the sharp decline of the proportion of sputum positive cases, which accounted for 30.5% of all cases in the 2000 survey but was reduced to 6.6% in the 2010 survey. The proportion of notified sputum cases out of all TB cases in Shandong also declined from 80.9% in 2005 to 64.6% in 2010 [19].

The prevalence rate of bacteriologically confirmed cases has reduced by 80% in the last decade in Shandong, compared with a national decline of 45% (from 216/ 100,000 in 2000 to 119/ 100,000 in 2010) [4]. The rapid decline of TB prevalence rate of bacteriologically confirmed cases in the recent decade may be attributed to China’s strengthened public health system following the outbreak of severe acute respiratory syndrome in 2003 [2]. Another reason may be due to improved reporting of TB cases in the online communicable disease reporting system, and the improved collaboration between public hospitals and TB dispensaries [20]. Other factors such as social economic development may also have played an important role in the reduction of TB prevalence, as found in a study of TB notification rates trends in 134 countries [21].

The adjusted prevalence rate of bacteriologically confirmed cases in Shandong was lower than the WHO estimates for China in 2010 [1]. But the national prevalence rates of bacteriologically confirmed cases, 119/100,000 in 2010 [4], was higher than the WHO estimate, 108/100,000, even the survey did not collect negative and extra-pulmonary TB cases. Vietnam reported similar findings in its 2006 survey [14]. One reason is that prevalence surveys results are based on active case finding while WHO estimates are based on notification rates from passive case finding. A re-evaluation of the reported TB prevalence in China is needed based on the recent survey.

CXRAY suggestive bacteriologically negative cases may be smear or culture negative TB cases if they had any TB symptoms, while some may be caused by suboptimal smear or culture. As reported in China’s previous surveys [3,22], including these cases as TB cases may result in an over-estimate of all pulmonary cases [23].

The survey revealed that over half of the TB patients were 65 years and older in Shandong, while the over 65’s were more likely to present with abnormal CXRAY and persistent cough. Similar trends have been documented in other developed cities such as Hong Kong and Singapore [24]. These high rates may reflect the higher TB rates in the past and decline in immunity in the over 65’s. How to treat elders with TB and other complications such as diabetes remains an ongoing challenge in China and similar settings.

The survey results can be generalized to the Shandong population of 94 million or similar international settings with middle income and middle TB prevalence levels. The patterns of the TB epidemic found in Shandong, i.e., the proportion of patients with symptoms, ratios between urban and rural areas, men and women, were similar to those found in the national survey [4]. However, the prevalence rates cannot be extrapolated to western provinces in China with a higher TB prevalence. For logistical reasons, the eligible population did not include adults staying in the sampled clusters less than 6 months, which was the same practice in the 2000 survey. However, short-term migrants may have a potentially higher prevalence of TB than the general population [25]. This may result in a lower estimate of the true prevalence rate. The survey did not collect social-economic indicators, smoking status and HIV status of all participants, so comparisons between TB cases and all non-TB patients are not available. However, the HIV prevalence in Shandong China is below 0.01%, and would not significantly alter the TB prevalence rate. In addition, the survey did not evaluate child TB and extra pulmonary TB. Discussions of using CXRAY as a screening tool was on the technical aspect, but not on costing side as we did not conduct any cost effectiveness analysis or the social willingness to pay for such a strategy in similar settings.

## Conclusions

This study has shown that the prevalence of bacteriologically confirmed TB in Shandong has reduced substantially over the last decade. Importantly, the majority of these cases did not present with persistent cough and the proportion of sputum positive cases has declined sharply. Further studies are recommended to assess the feasibility of adopting CXRAY in the existing health care services to detect TB cases and the cost effectiveness of such intervention.

## Abbreviations

TB: Tuberculosis; CXRAY: Chest X-Ray; WHO: The World Health Organization; IRB: The Institutional Review Board.

## Competing interests

The authors declare that they have no competing interests.

## Authors’ contributions

XW, XZ, FL, ZL, JY and GZ have designed the research and its tools. XW and XZ oversaw the study and manuscript development. The manuscript was written by XW, XZ, JW and RB. XZ, FL, HZ, ZL and XW conducted the survey. JY, GZ and HZ conducted literature review and data analysis. BZ provided statistical support on data interpretation. SG provided significant comments on revision of the paper. All authors read and approved the manuscript. XZ had full access to all the data in the study and takes responsibility for the integrity of the data and the accuracy of the data analysis.

## Pre-publication history

The pre-publication history for this paper can be accessed here:

http://www.biomedcentral.com/1471-2334/14/21/prepub
